# Long non-coding RNA00544 serves as a potential novel predictive and prognostic marker for HR+ HER2− subtype breast cancer

**DOI:** 10.1038/s41598-017-11066-7

**Published:** 2017-09-28

**Authors:** Lei Liu, Yayun Chi, Jiajian Chen, Jingyan Xue, Linlin Deng, Naisi Huang, Jianghua Shao, Jiong Wu

**Affiliations:** 1 Department of Breast Surgery, Fudan University Shanghai Cancer Center, Shanghai, 200032 China; 2 Department of General Surgery, Second Affiliated Hospital of Nanchang University, Nanchang, 330006 China; 3 Department of Oncology, Fudan University, Shanghai Medical College, Shanghai, 200032 China; 4 Collaborative Innovation Center for Cancer Medicine, Shanghai, China

## Abstract

Luminal breast cancers (BC) account for majority of breast cancer. Due to its heterogeneity and the development of treatment resistance, luminal BC patients can vary substantially. Long noncoding RNAs (lncRNAs), as we known, is involved in breast cancer progression. Here, we aim to identify the lncRNAs which are involved in the particular type luminal BC progression. By Gene Chips analysis, we found a novel lncRNA00544, which was highly expressed in the metastatic axillary nodes compared with corresponding luminal BC tissues (fold change = 2.26, P = 0.043). This result was confirmed in luminal BC cell lines (p = 0.0113) and 49 paired breast cancer samples compared with in corresponding controls (p = 0.011). Furthermore, Kaplan–Meier survival curves of 373 breast cancer patients indicated that disease-free survival was significantly poor in breast cancer patients with high lncRNA00544 expression (p < 0.001). Univariate and multivariate Cox regression analyses showed that lncRNA00544 was a significant independent prognostic biomarker in luminal BC patients. Further analysis showed that the prognosis of high lncRNA00544 expression in breast cancer patients was actually related to HR + HER2− subtype. Together, our studies indicate that lncRNA00544 may represent a novel predictive and prognostic indicator in luminal BC patients.

## Introduction

Breast cancer (BC) is the most common cancer of women worldwide, and approximately 60–75% of cases are luminal tumors^1,2^. Luminal BC is a highly heterogeneous disease characterized by hormone receptor positivity (HR+) and can be further classified as luminal A and luminal B based on human epidermal growth factor receptor-2 (HER2) and Ki67 status^3^. Luminal BC can also be termed as HER2-negative (HR+/HER2−) and HER2-positive (HR+/HER2+), the latter of which is more aggressive and is treated with anti-HER2 therapy. Although nearly all women with luminal tumors show highly effective responses to endocrine therapy, some show substantial variation in their clinical course and treatment response^4^, such as early or late relapses and metastasis^5^ resulting in relatively worse prognosis. Therefore, it is extremely important to identify novel molecular biomarkers that can predict the progression and prognosis of luminal BC, and identify patients for whom adjuvant endocrine treatment might be beneficial.

Long non-coding RNAs (lncRNAs) are a subtype of non-coding RNAs composed of more than 200 nucleotides with little or no protein-coding capacity^6^. Numerous studies indicate that lncRNAs are involved in many biological and pathological processes, including chromatin modification, transcriptional regulation, and post-transcriptional regulation^7,8^. Deregulation of lncRNAs have been proven to be important in various human diseases, particularly in human cancer^9^. Recent studies have demonstrated that dysregulated lncRNAs play potential roles as biomarkers in the diagnosis and prognosis of many cancer types^10–13^, including breast cancer^14^. Many differentially expressed lncRNAs including circulating lncRNA and lncRNA signatures, such as H19^15^, lncRNA HOX antisense intergenic RNA (HOTAIR)^16^, 12-lncRNA signature^17^ and breast cancer anti-estrogen resistance 4 (BCAR4)^18^ have been detected in breast cancer plasma, tissues and cell lines. Notably, lncRNAs have been found that they display tumor subtype specific expression in breast cancer where lncRNA expression alone is sufficient to distinguish samples based on hormone status and molecular intrinsic subtype^19,20^.

Accumulating evidence suggests that lncRNAs are associated with metastasis and prognosis of estrogen receptor-positive (ER+) breast cancer^21,22^. For example, overexpression of a specific transcribed-ultra conserved region (T-UCR) named uc.63, one of a new class of lncRNAs, is associated with worse prognosis in patients with the luminal A subtype of breast cancer^23^. HOTAIR is overexpressed in ER+ breast cancer compared with ER− tumors, and serves as an independent biomarker of metastasis in ER-positive breast cancer^24^. Overexpression of metastasis associated in lung adenocarcinoma transcript 1 (MALAT1) is associated with poor prognosis in tamoxifen-treated ER+ breast cancer patients, and might be considered as a potential biomarker to predict endocrine treatment sensitivity^25^. However, these previous reports focused on ER+ breast cancer. The potential correlation between lncRNAs and clinical outcome in HR+ breast cancer patients, especially in the HR+/HER2− subtype, remains unknown.

In this study, we investigated differentially expressed lncRNAs using Affymetrix Human Transcriptome Array 2.0 (HTA 2.0) Gene Chips for five luminal BC tissue samples and matched metastatic axillary nodes. Based on the results of the array analysis, we focused on a novel lncRNA00544 (ENST00000544591, 4687 nucleotides; chromosome 12 (+): 10705962–10710648), which was highly expressed in metastatic axillary nodes compared with BC tissue samples. To determine whether this novel lncRNA might be a potential biomarker for the progression of luminal BC, the expression of lnc00544 in breast cancer tissues and axillary nodes was compared by quantitative real-time polymerase chain reaction (qRT-PCR). In addition, we analyzed its association with clinical and pathological features of breast cancer. Finally, the predictive value of lnc00544 expression for prognosis in HR+/HER2− breast cancer was evaluated by Kaplan-Meier and Cox regression analysis.

## Results

### LncRNA00544 is upregulated in luminal breast cancer

To assess possible relationships between lncRNAs and prognosis of luminal BC, we selected differentially expressed lncRNAs from the Affymetrix Human Transcriptome Array 2.0 (HTA 2.0) Gene Chips of five luminal BC tissue samples and matched metastatic axillary nodes (see Supplementary Fig. S1). From 5 paired samples we found that there were 45 up-regulated lncRNAs (fold change ≥1.5 and P < 0.05) and 153 downregulated lncRNAs (fold change ≥1.5 and P < 0.05). From up-regulated lncRNAs (fold change ≥2.0 and P < 0.05) (see Supplementary Table S1), we selected a novel lncRNA00544 (ENST00000544591, 4,687 nucleotides; chromosome 12 (+): 10705962–10710648) that was highly expressed in metastatic axillary nodes compared with BC tissue samples (fold change = 2.26, p = 0.043).

**Figure 1:**
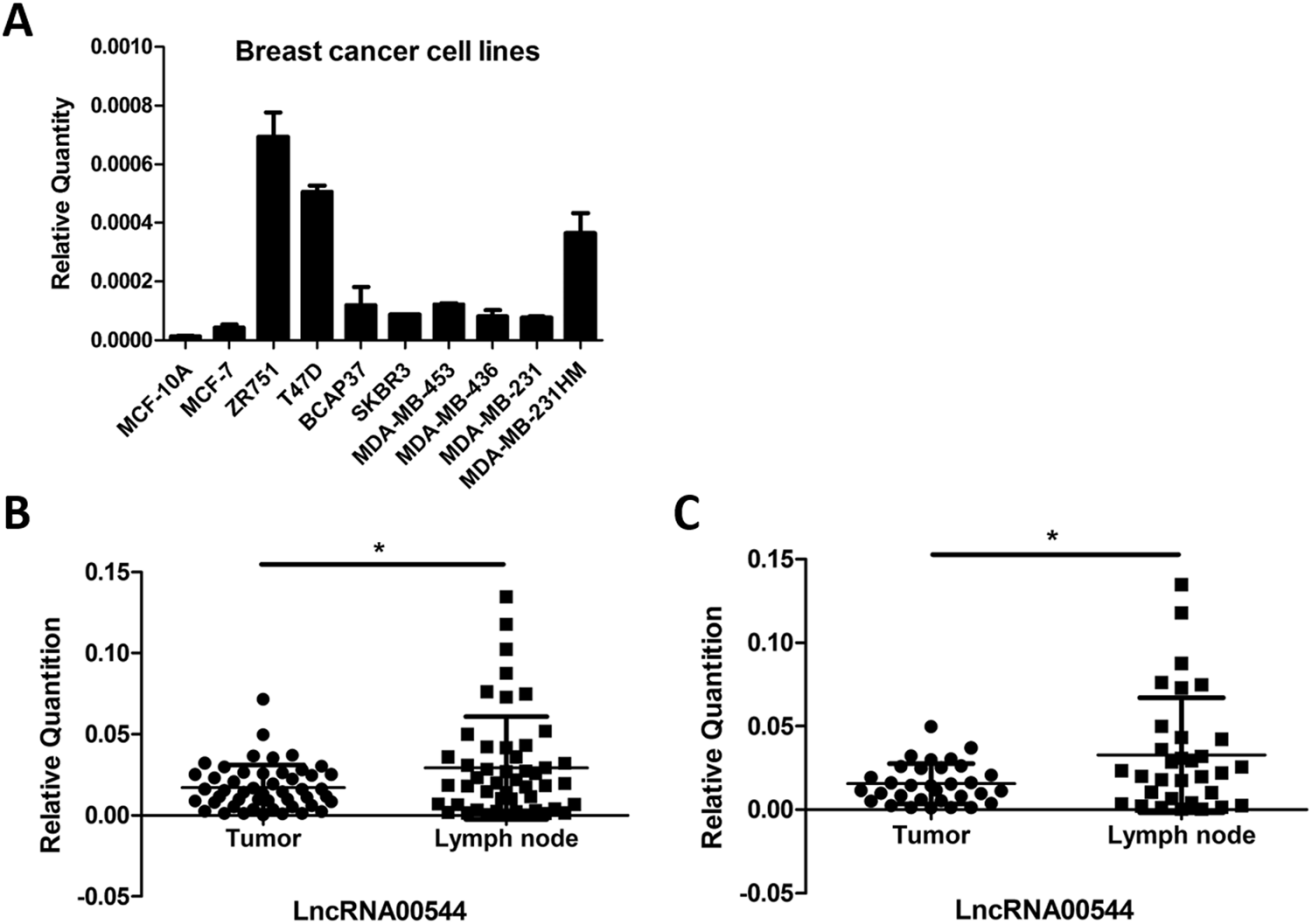
LncRNA00544 expression in luminal breast cancer. (**A**) Expression of lncRNA00544 in nine breast cancer cell lines (MCF-7, ZR751, T47D, BCAP37, SKBR3, MDA-MB-453, MDA-MB-436, MDA-MB-231, and MDA-MB-231HM) and normal breast cells (MCF10A) by qPCR. *GAPDH* was used as an internal control. (**B**) Comparison of lncRNA00544 expression in 49 breast cancer tissues and matched metastatic axillary nodes by qPCR. (**C**) Expression of lncRNA00544 in 32 pairs of luminal BC tissues and matched metastatic axillary nodes by qPCR. *p < 0.05, **p < 0.01.

To confirm the differential expression of lncRNA00544 in breast cancer, we showed that the expression of lncRNA00544 was increased in all BC cell lines compared with a normal breast cell line (MCF10A) (p < 0.05, Fig. 1A). More significantly, lncRNA00544 expression in luminal BC cell lines (MCF-7, ZR751, T47D) was significantly higher than in cell lines of other subtypes (p = 0.0113). Moreover, lncRNA00544 expression in MDA-MB-231HM, which is a highly lung metastatic BC cell line, was much higher than that in its parental cancer cell line MDA-MB-231 (p = 0.002). The relative expression of lncRNA00544 was further analyzed in 49 paired breast cancer tissues and matched metastatic axillary nodes by qRT-PCR normalized to *GAPDH*. LncRNA00544 expression was significantly higher in metastatic axillary nodes compared with breast cancer tissue (p = 0.0186, Fig. 1B), and especially in the luminal BC subtype (p = 0.011, Fig. 1C).

### Relationship between lncRNA00544 expression and clinical features

To further identify the significance of lncRNA00544 expression in breast cancer, we examined the relationship between lncRNA00544 expression and clinical features. Patients were categorized as low or high expression according to the median level of lncRNA00544 expression in the breast tumors. The cutoff value of lncRNA00544 high and lncRNA00544 low groups for this study was determined by the median. Of the 373 breast cancer patients, 186 cases were classified as low lncRNA00544, and the other 187 as high lncRNA00544. As shown in Table 1, high expression of lncRNA00544 was associated with positive Ki67 expression (p = 0.055). However, there was no significant correlation between lncRNA00544 and any other clinicopathologic parameters (p > 0.05).

**Table 1:** Relationship between lncRNA00544 expression and clinicopathologic features in BC patients.

Characteristics	LncRNA00544		N	χ^2^	P-value
	Low	High			
Age (years)					
≤50	91 (48.9%)	81 (43.3%)	172	1.181	0.300
>50	95 (51.1%)	106 (56.7%)	201		
BMI (kg/m^2^)					
<25	131 (72.0%)	136 (73.5%)	267	0.109	0.815
≥25	51 (28.0%)	49 (26.5%)	100		
Menopausal status					
Pre	88 (47.6%)	81 (43.5%)	169	0.604	0.466
Post	97 (52.4%)	105 (56.5%)	202		
MT Family Hix					
No	175 (94.1%)	174 (93.5%)	349	0.046	1.000
Yes	11 (5.9%)	12 (6.5%)	23		
Tumor size (cm)*					
≤2	69 (37.1%)	59 (31.6%)	128	1.272	0.277
>2	117 (62.9%)	128 (68.4%)	245		
TNM stage					
I-II	140 (75.3%)	134 (71.7%)	274	0.624	0.482
III	46 (24.7%)	53 (28.3%)	99		
Lymph node status					
Negative	88 (47.8%)	95 (50.8%)	183	0.329	0.604
Positive	96 (52.2%)	92 (49.2%)	188		
Tumor grade					
I-II	91 (53.5%)	87 (49.2%)	178	0.665	0.453
III	79 (46.5%)	90 (50.8%)	169		
HR status					
Negative	73 (39.2%)	92 (49.2%)	165	3.743	0.061
Positive	113 (60.8%)	95 (50.8%)	208		
HER-2 status					
Negative	103 (60.2%)	107 (60.5%)	210	0.002	1.000
Positive	68 (39.8%)	70 (39.5%)	138		
Ki67 status					
Negative	73 (50.0%)	67 (39.2%)	140	3.738	0.055
Positive	73 (50.0%)	104 (60.8%)	177		
LVI status					
Negative	106 (59.6%)	100 (54.1%)	206	1.117	0.340
Positive	72 (40.4%)	85 (45.9%)	157		

Abbreviations: BMI: body mass index; MT Family Hix: malignant tumor family history; HR: hormone receptor, estrogen receptor or/and progesterone receptor; HER2: human epidermal growth factor receptor-2; LVI: lymphovascular invasion. *Only the size of invasive tumor is included.

### LncRNA00544 overexpression is a poor prognostic factor for breast cancer patients

**Figure 2:**
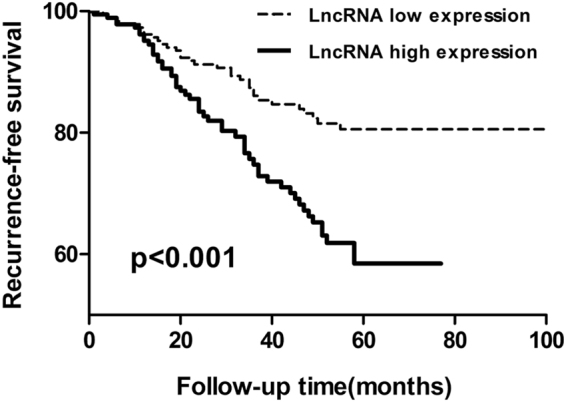
Kaplan–Meier analysis of disease-free survival of breast cancer patients

To assess whether lncRNA00544 expression correlated with prognosis in patients with breast cancer, we analyzed the follow-up cohort of 373 patients for DFS. As shown in Fig. 2, patients with high lncRNA00544 expression showed significantly shorter DFS than those with low expression (p < 0.001). Univariate Cox proportional hazards regression analysis of DFS demonstrated that larger tumor size (HR = 2.22; 95% CI: 1.304–3.781; p = 0.003), TNM stage (HR = 3.435; 95% CI: 2.239–5.27; p < 0.001), lymph node (HR = 3.224; 95% CI: 1.967–5.284; p < 0.001), Ki67 status (HR = 1.775; 95% CI: 1.098-2.87; p = 0.019), lymphovascular invasion (LVI) status (HR = 3.236; 95% CI: 2.039–5.135; p < 0.001), and high lncRNA00544 expression (HR = 2.284; 95% CI: 1.463–3.565; p < 0.001) were distinctively linked with the prognosis of breast cancer (Table 2). Furthermore, multivariate Cox regression analysis revealed that breast cancer patients with high lncRNA00544 expression had significantly worse prognosis than those with low expression levels (HR = 2.293; 95% CI: 1.343–3.915; p = 0.002, Table 3). This indicated that lncRNA00544 was an independent prognostic indicator for breast cancer patients.

**Table 2:** Univariate regression model of prognostic covariates in breast cancer patients.

Variable		HR	95.0%Cl		P-value
			Low	High	
Age (years)	≤50 vs. >50	0.79	0.514	1.212	0.282
BMI (kg/m^2^)	<25 vs. ≥25	1.232	0.77	1.972	0.385
Menopausal	Pre vs. Post	0.953	0.621	1.462	0.824
MT Family Hix	Negative vs. Positive	0.478	0.151	1.514	0.21
Tumor size (cm)	≤2 vs.>2	2.22	1.304	3.781	**0.003**
TNM stage	I-II vs. III	3.435	2.239	5.27	**<0.001**
Lymph node	Negative vs. Positive	3.224	1.967	5.284	**<0.001**
Tumor grade	I-II vs. III	1.298	0.832	2.026	0.251
HR status	Negative vs. Positive	1.009	0.646	1.576	0.968
HER-2 status	Negative vs. Positive	0.74	0.467	1.172	0.2
Ki67 status	Negative vs. Positive	1.775	1.098	2.87	**0.019**
LVI status	Negative vs. Positive	3.236	2.039	5.135	**<0.001**
LncRNA00544	High vs. Low	2.284	1.463	3.565	**<0.001**

Abbreviations: HR: hazard ratio; CI: confidence interval; BMI: body mass index; MT Family Hix: malignant tumor family history; TNM: Tumor Node Metastasis; LVI: lymphovascular invasion; HR+: hormone receptor, estrogen receptor or/and progesterone receptor positive; HER2−: human epidermal growth factor receptor-2 negative. Bold font indicates p < 0.05.

**Table 3:** Multivariate analysis of clinicopathologic factors for recurrence-free survival in breast cancer patients.

Variable		HR	95.0%Cl		P-value
			Low	High	
Tumor size (cm)	≤2 vs. >2	1.57	0.83	2.969	0.165
Lymph node	Negative vs. Positive	1.579	0.676	3.689	0.291
TNM stage	I-II vs. III	1.805	0.952	3.42	0.07
HR status	Negative vs. Positive	1.044	0.584	1.866	0.885
Tumor grade	I-II vs. III	0.962	0.551	1.681	0.893
Ki67 status	Negative vs. Positive	1.678	0.946	2.977	0.077
LVI status	Negative vs. Positive	1.462	0.711	3.006	0.302
LncRNA00544	High vs. Low	2.293	1.343	3.915	**0.002**

Abbreviations: HR: hazard ratio; CI: confidence interval; TNM: Tumor Node Metastasis; LVI: lymphovascular invasion; HR+: hormone receptor, estrogen receptor or/and progesterone receptor positive; HER2−: human epidermal growth factor receptor-2 negative. Bold font indicates p < 0.05.

### LncRNA00544 expression is correlated with prognosis in patients with HR + HER2**−** breast cancer

**Figure 3:**
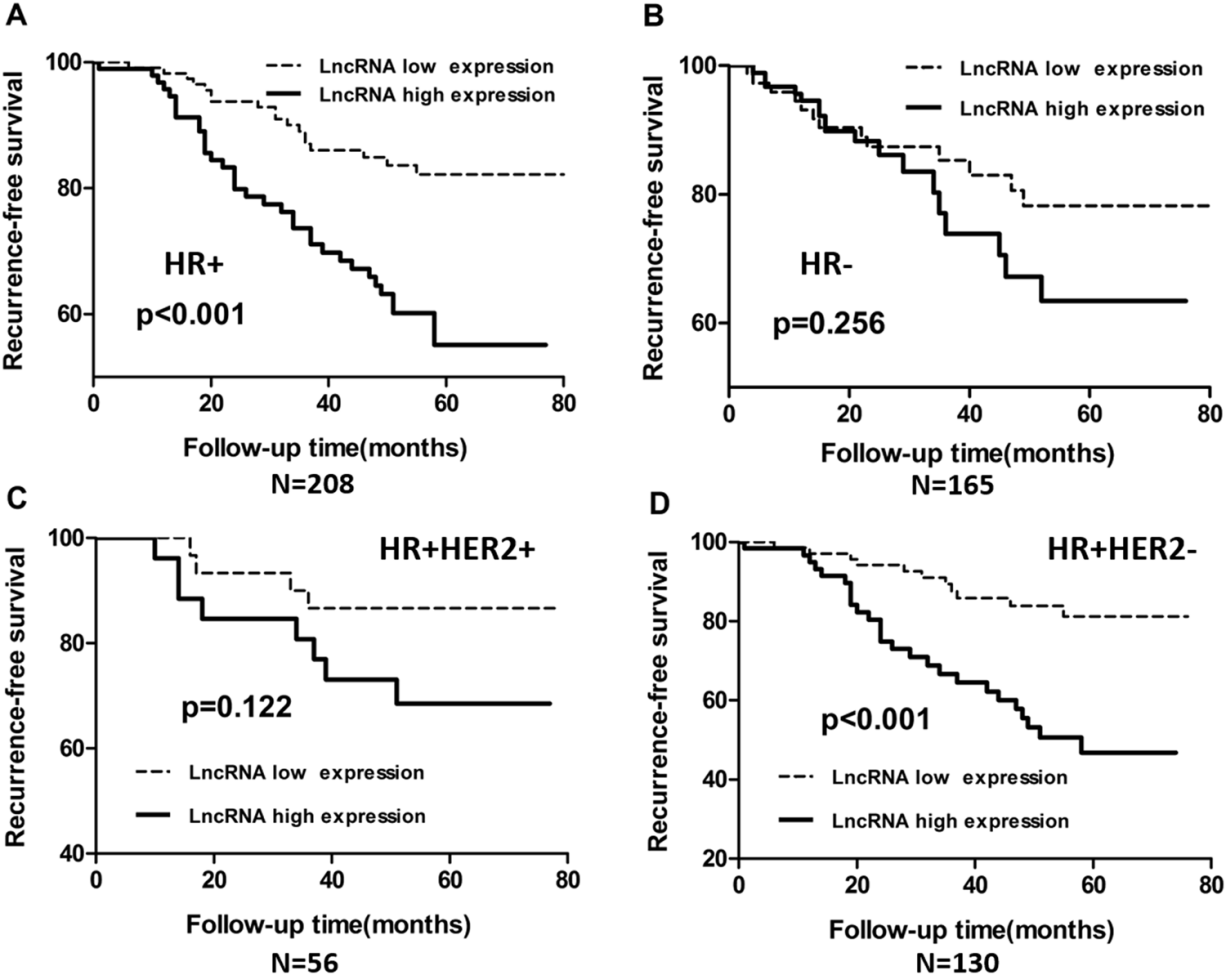
Kaplan–Meier analysis of disease-free survival of breast cancer patients according to HR and HER2 status. Kaplan-Meier survival analysis of DFS rate in patients with (**A**) HR + BC, (**B**) HR− BC, (**C**) HR + HER2 + BC, (**D**) HR + HER2− BC.

The patients were then divided into HR positive (HR+) and HR negative (HR−) subtypes based on ER or/and PR expression. Kaplan–Meier survival curves indicated that the prognostic value of lncRNA00544 for DFS was of high significance among HR+ patients (p < 0.001, Fig. 3A), but displayed no significant difference in the HR− BC group (p = 0.256, Fig. 3B). Further analysis revealed that high lncRNA00544 expression was significantly associated with prognosis of the HER2−/HR + BC group (p < 0.001, Fig. 3D), but not with that of the HER2+/HR + BC group (p = 0.122, Fig. 3C). In order to verify the results, we analyzed disease-free survival of breast cancer patients according to HER2 status at the same time (see Supplementary Fig. S2) and the results of the analysis show the same trend as before that lncRNA00544 expression is correlated with prognosis in patients with HER2− HR+ breast cancer.

In the HR+/HER2− BC group, univariate Cox proportional hazards analysis revealed that lncRNA00544 expression (HR = 3.573; 95% CI: 1.762–7.242; p < 0.001), TNM stage (HR = 3.273; 95% CI: 1.705–6.281; p < 0.001), Ki67 (HR = 2.508; 95% CI: 1.3–4.84; p = 0.006), and LVI status (HR = 2.423; 95% CI: 1.226–4.788; p = 0.011) were prognostic indicators (Table 4). Patients with high lncRNA00544 expression showed a higher likelihood of occurrence of disease events (HR = 3.573; 95% CI: 1.762–7.242; p < 0.001) in univariate analysis and maintained the same trend in multivariate analysis (HR = 2.752; 95% CI: 1.274–5.944; p = 0.01; Table 5). In addition, Ki67 status also can serve as an independent prognostic indicator for poor DFS among HR + HER2− BC patients (HR = 2.262; 95% CI: 1.071–4.776; p = 0.032, Table 5). This indicated that lncRNA00544 was an independent prognostic indicator for breast cancer patients with HR + HER2− expression.

**Table 4:** Univariate regression model of prognostic covariates in HR + HER2− BC Patients.

Variable		HR	95.0%Cl		P-value
			Low	High	
Age (years)	≤50 vs. >50	0.897	0.465	1.732	0.747
BMI (kg/m^2^)	<25 vs. ≥25	1.099	0.538	2.244	0.796
Menopausal	Pre vs. Post	0.955	0.498	1.834	0.89
MT Family Hix	Negative vs. Positive	1.058	0.254	4.402	0.938
Tumor size (cm)	≤2 vs. >2	2.017	0.921	4.418	0.079
TNM stage	I-II vs. III	3.273	1.705	6.281	**<0.001**
Lymph node	Negative vs. Positive	3.076	1.35	7.006	0.007
Tumor grade	I-II vs. III	1.906	0.973	3.731	0.06
Ki67 status	Negative vs. Positive	2.508	1.3	4.84	**0.006**
LVI status	Negative vs. Positive	2.423	1.226	4.788	**0.011**
LncRNA00544	High vs. Low	3.573	1.762	7.242	**<0.001**

Abbreviations: HR: hazard ratio; CI: confidence interval; BMI: body mass index; MT Family Hix: malignant tumor family history; TNM: Tumor Node Metastasis; LVI: lymphovascular invasion; HR+: hormone receptor, estrogen receptor or/and progesterone receptor positive; HER2−: human epidermal growth factor receptor-2 negative. Bold font indicates p < 0.05.

**Table 5:** Multivariate analysis of clinicopathologic factors for recurrence-free survival in HR + HER2− BC patients.

Variable		HR	95.0%Cl		P-value
			Low	High	
Tumor size (cm)	≤2 vs. >2	1.107	0.434	2.823	0.831
Lymph node	Negative vs. Positive	1.805	0.545	5.98	0.334
TNM stage	I-II vs. III	1.654	0.636	4.302	0.302
Ki67 status	Negative vs. Positive	2.262	1.071	4.776	**0.032**
Tumor grade	I-II vs. III	1.077	0.48	2.418	0.857
LVI status	Negative vs. Positive	0.967	0.304	3.078	0.955
LncRNA00544	High vs. Low	2.752	1.274	5.944	**0.01**

Abbreviations: HR: hazard ratio; CI: confidence interval; TNM: Tumor Node Metastasis; LVI: lymphovascular invasion; HR+: hormone receptor, estrogen receptor or/and progesterone receptor positive; HER2−: human epidermal growth factor receptor-2 negative. Bold font indicates p < 0.05.

## Discussion

Here, we reported for the first time that a novel lncRNA00544 selected from Affymetrix Gene Chips of five luminal BC tissue samples and their matched metastatic axillary nodes was significantly associated with progression and prognosis of HR + HER2− breast cancer.

It has been reported that luminal breast cancer represents almost two-thirds of all breast cancer cases^26^. For these patients, endocrine therapies are conventionally recommended in both adjuvant and recurrent settings^27^. However, 40% to 50% of luminal breast cancer patients inevitably experience relapse, even decades after surgery^28,29^. Consistent with their high prevalence among breast cancers, luminal tumors contribute to most breast cancer deaths. Therefore, novel and specific biomarkers for significant clinical progression and prognosis of luminal BC are urgently needed.

Accumulating evidence has demonstrated that dysregulated lncRNAs play a crucial role in luminal BC progression and metastases^30^. For instance, DSCAM-AS1, one of the Apo-ERα-Regulated lncRNAs (AER-lncRNAs) is expressed in breast cancer with ER-alpha positive (ERα +) status and correlates inversely with epithelial-to-mesenchymal transition (EMT), and was confirmed as a tumor suppressor in luminal-like breast cancer^31^. The lncRNA BC200, also called brain cytoplasmic RNA 1 (BCYRN1), is upregulated in luminal breast cancer. Its expression could be induced by estrogen signaling, therefore BC200 may serve as a prognostic marker in estrogen-dependent breast cancer^32^. The lncRNA HOTAIR, which is associated with reprogramming of the chromatin state and induction of cancer metastasis^33^, is a powerful predictor of poor clinical outcome, especially in ER-positive breast cancer^24,34^. However, the potential correlation between lncRNAs and clinical outcome in patients with HR + HER2− breast cancer remains unknown.

In the present study, we identified a novel lncRNA00544 from gene chips of luminal BC tissues. Our results showed that lncRNA00544 expression is upregulated in luminal and metastatic BC cell lines compared with a normal breast cell line, as well as in metastatic axillary nodes compared with pair-matched tumor tissue, especially in patients with luminal BC, which was concordant with the results from gene chips. Moreover, Kaplan–Meier survival curves showed that patients with high lncRNA00544 expression had significantly poor DFS compared with the low lncRNA00544 expression group, and that these differences existed in HR+ cancers but not in HR− tumors. These data suggest that dysregulation of lncRNA00544 is a novel biomarker associated with poor progression of luminal BC.

Based on HER2 expression, luminal BCs can be further classified as HR + HER2− and HR + HER2+ subtypes. HR + HER2− represents the majority (approximately 73%) of breast cancer patients^35^. Although these patients can benefit from endocrine therapy, because of its high frequency HR + HER2− BC accounts for more recurrences and deaths than other breast cancer subtypes^36^. HR + HER2− breast cancers have different biology from HR+ HER2+ breast cancers^37^. Because of the heterogeneity of luminal BCs and based on reports that specific expression of lncRNAs can be a useful tool to distinguish between the various breast cancer subtypes^38^, we further classified the HR + BC group according to lncRNA00544 expression and reanalyzed the data. Our results demonstrated that high lncRNA00544 expression was significantly linked with the prognosis of HER2− patients, but was not statistically significant in the HER2+ group. Univariate analysis and multivariate analysis indicated that lncRNA00544 might be an independent prognostic indicator in addition to Ki67 status, and upregulated lncRNA00544 was correlated with unfavorable DFS in HR + HER2− BC patients. To the best of our knowledge, this is the first report of the involvement of lncRNA00544 in the progression and prognosis of HR + HER2− breast cancer.

It has been reported that lncRNAs can act as cis-regulators of their genetically neighboring protein-coding genes as or trans-regulators of distant protein-coding genes in cancer^39,40^. Through the University of California Santa Cruz (UCSC) genome browser (http://genome.ucsc.edu/) we identified that serine threonine tyrosine kinase 1 (*STYK1*; chr12: 10,771,538–10,826,891), which is known to be involved in tumor metastasis by activating of phosphoinositide 3-kinase (PI3K)/AKT signaling pathways^41^, is located 39,779 bp downstream of lncRNA00544. PI3K/AKT pathway alterations have been frequently reported in the luminal breast cancer subtypes^42^, and especially in HR + HER2− breast cancer^43^, suggesting crosstalk between ER and PI3K/AKT. Based on these data, we speculated that lncRNA00544 might in part function as a tumor oncogene in HR + HER2− breast cancer via the STYK1 gene and the PI3K/AKT pathway. However, elucidation of the exact mechanism requires further experimental studies.

Considering all of the above data, this is the first study to present the novel lncRNA00544 with increased expression in luminal BC cell lines and metastatic axillary nodes samples. In addition, our results showed that dysregulated lncRNA00544 was a significant independent prognostic biomarker in patients with the HR + HER2− subtype of breast cancer patients. Taken together, these results suggest that lncRNA00544 may represent a novel prognostic indicator and a target for gene therapy in HR + HER2− breast cancer. These data provide essential information for individualized prognosis and treatment decisions in these patients.

## Methods

### Tissue samples and clinical data collection

Our study was approved by Medical Ethics Committee of Shanghai Cancer Center FUDAN University (NO. 050432-4-1212B). Human breast cancer tissue samples were obtained from the Department of Breast Surgery in Fudan University Shanghai Cancer Center after obtaining informed consent from patients diagnosed with breast cancer. In addition to 373 primary breast cancer samples of stage I to III invasive ductal carcinoma (collected postoperatively from February 2007 to December 2012), a cohort of 49 breast cancer tissues and pair-matched metastatic axillary nodes were resected during surgery and immediately frozen in liquid nitrogen for subsequent total RNA extraction. Patients who received chemotherapy or radiotherapy before sample collection or had metastatic disease were excluded.

Clinicopathologic features were mainly collected from medical records, pathology reports, and personal interviews, including baseline data for patients, information on surgery, pathologic data, and follow-up of the tumor. The clinical staging criteria were assessed according to the American Joint Committee on Cancer TNM classification (2010). Pathologic diagnosis and ER, PR, HER2, and Ki67 status were determined by at least two academic pathologists according to the World Health Organization (WHO) classification and American Society for Clinical Oncology (ASCO) guidelines. We confirm that all methods were performed in accordance with the relevant guidelines and regulations.

### LncRNA microarray analysis

Total RNA was extracted from 5 luminal BC tissue samples and matched metastatic axillary nodes. Sample preparation and microarray hybridization were performed based on the standard Affymetrix HTA 2.0 chips protocol. Affymetrix^®^ GeneChip Command Console (AGCC) installed in GeneChip^®^ Scanner 3000 7G, was used to scan genechips, save the image data and compute the probe intensity data. Quantile normalization was performed by the Expression Console Software and subsequent data was processed by Affymetrix^®^ Transcriptome Analysis Console (TAC) software.The microarray was performed by Shanghai Gminix Biological Information Company (Shanghai, China). Differentially expressed lncRNAs with statistical significance (P < 0.05; fold change >1.5) were identified by comparing the normalized expression levels in BC tissue samples and matched metastatic axillary nodes with random variance model t test.For the Gene Ontology enrichment and Kyoto Encyclopedia of Genes and Genomes(KEGG) pathway analysis, the Integrated Discovery (DAVID) webserver (http://david.ncifcrf.gov/) was used.

### Cell lines and culture conditions

Nine breast cancer cell lines (MCF-7, ZR751, T47D, BCAP37, SKBR3, MDA-MB-453, MDA-MB-436, MDA-MB-231, MDA-MB-231HM) and a normal breast cell line (MCF-10A) were obtained from the cell bank of our laboratory. MDA-MB-231 and MDA-MB-231HM cells were cultured in F15 medium (HyClone, USA). MCF-7, ZR751, T47D, BCAP37, SKBR3, MDA-MB-453, and MDA-MB-436 cells were cultured in DMEM medium (Hyclone, USA). MCF10A cells were cultured in F12/DMEM 1:1 medium (Gibco, USA). All cells were cultured with 10% fetal bovine serum (Gibco, USA), 100 units/ml penicillin (Life technology, USA), and 100 g/ml streptomycin (Life technology, USA) at 37 °C in a humidified CO_2_ (5%) atmosphere.

### RNA extraction and quantitative RT-PCR

Quantitative real-time polymerase chain reaction (qRT-PCR) was applied to validate the expression level of chosen lncRNA ENST00000544591, which we named as lncRNA00544. Total RNA was extracted from tissue samples and cell lines using TRIzol reagent (Invitrogen, USA) according to the manufacturer’s instructions. After converting total RNA of lncRNA00544 to cDNA in a reverse transcription (RT) reaction, qPCR was performed using SYBR Select Master Mix (Takara, Japan) on an ABI 7900 system (Applied Biosystems, USA). Melting curve analysis was used to monitor the specificity of the PCR result. Relative expression of lncRNA00544 compared with *GAPDH* was determined using the comparative delta-delta CT method (2-delta Ct). All reactions were performed in triplicate. The primers of GAPDH and lncRNA00544 were synthesized by Sangon Biotech (Shanghai, China). The primer sequences were as follows:

lncRNA00544 forward: 5′-ACCTTTGAACACGATGGGACA-3′;

lncRNA00544 reverse: 5′-TCTCCTCGGGGGAGCTTAAA-3′.

### Statistical and bioinformatics analysis

All data were analyzed for statistical significance using SPSS 21.0 software (SPSS Inc., Chicago, IL, USA) and GraphPad Prism 5 (Graphpad Software Company, USA). An unpaired t-test was used to analyze the difference between breast cancer cell lines. A paired Wilcoxon signed rank test was used to examine lnc00544 expression in breast cancer tissues versus pair-matched metastatic axillary nodes. The Pearson Chi-square test was applied to the examination of correlation between lncRNA00544 expression and clinicopathologic characteristics. The interval from the date of initial surgery to progression (local and/or distal tumor recurrence) was defined as disease-free survival (DFS). DFS was calculated by the Kaplan–Meier method and the log rank test. Cox univariate and multivariate regression analyses were performed to evaluate prognostic significance of each parameter in patients with luminal subtype breast cancers. Adjusted hazard ratios (HRs) with 95% confidence intervals (CIs) were calculated using Cox proportional hazards modeling. Adjusted odds ratios (ORs) with 95% CIs were determined using multivariate logistic regression. All tests were two-sided, and p < 0.05 was regarded statistically significant. Death from another disease was regarded as censored.

## Electronic supplementary material

Supplementary Information
