# Supplementary material for: Long non-coding RNA00544 serves as a potential novel predictive and prognostic marker for HR+ HER2− subtype breast cancer
Source: Sci Rep. 2017 Sep 28;7:12382. doi: 10.1038/s41598-017-11066-7 (PMC5620366; doi:10.1038/s41598-017-11066-7)
Supplement: Supplementary file 1 — Supplementary Information [file 41598_2017_11066_MOESM1_ESM.doc]

**Long non-coding RNA00544 serves as a potential novel predictive and prognostic marker for HR+HER2− subtype breast cancer**

Lei Liu^1,2,+^, Yayun Chi^2,3,+^, Jiajian Chen^2,3^,, Jingyan Xue^2,3^,, Linlin Deng ^1,2^, Naisi Huang^2,3^, Jianghua Shao^1,*^, Jiong Wu^2,3,4,*^

^1^ Department of General Surgery, Second Affiliated Hospital of Nanchang University, Nanchang, 330006, China

^2^ Department of Breast Surgery, Fudan University Shanghai Cancer Center, 200032, China

^3^Department of Oncology, Fudan University, Shanghai Medical College, Shanghai, 200032, China

^4^Collaborative Innovation Center for Cancer Medicine, China

^+^These authors contributed equally to this work

^*^Corresponding authors: Jiong Wu, e-mail: [wujiong1122@vip.sina.com;](mailto:wujiong1122@vip.sina.com;) Jianghua Shao, e-mail: [shao5022@163.com](mailto:shao5022@163.com)

Supplementary Information

Figure.S1: Transcript expression changes determined by the GeneChip®Human Transcriptome Array. (A) Heat map of expression changes on upregulated lncRNAs (n=45); the target lncRNA lncRNA00544 is one of upregulated lncRNAs; (B) Heat map of expression changes on downregulated lncRNAs (n=153). Data analysis was performed by using the following parameters: one-way between-subject ANOVA (unpaired), fold change (linear) < −1.5 or fold change (linear) > 1.5, and ANOVA p value (condition pair) < 0.05. Abbreviations: BC: breast cancer tissue; Ctrl: matched metastatic axillary node tissue.

**A**


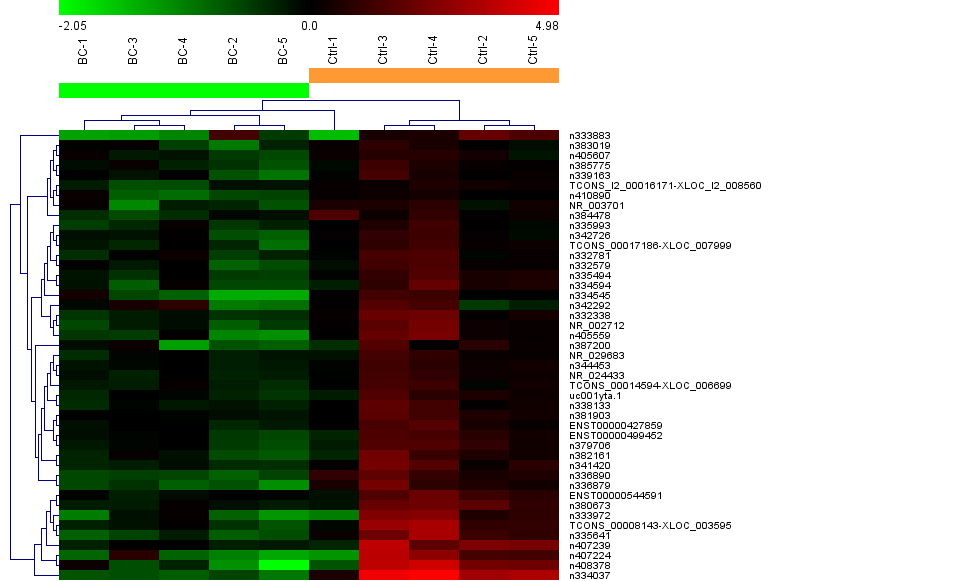


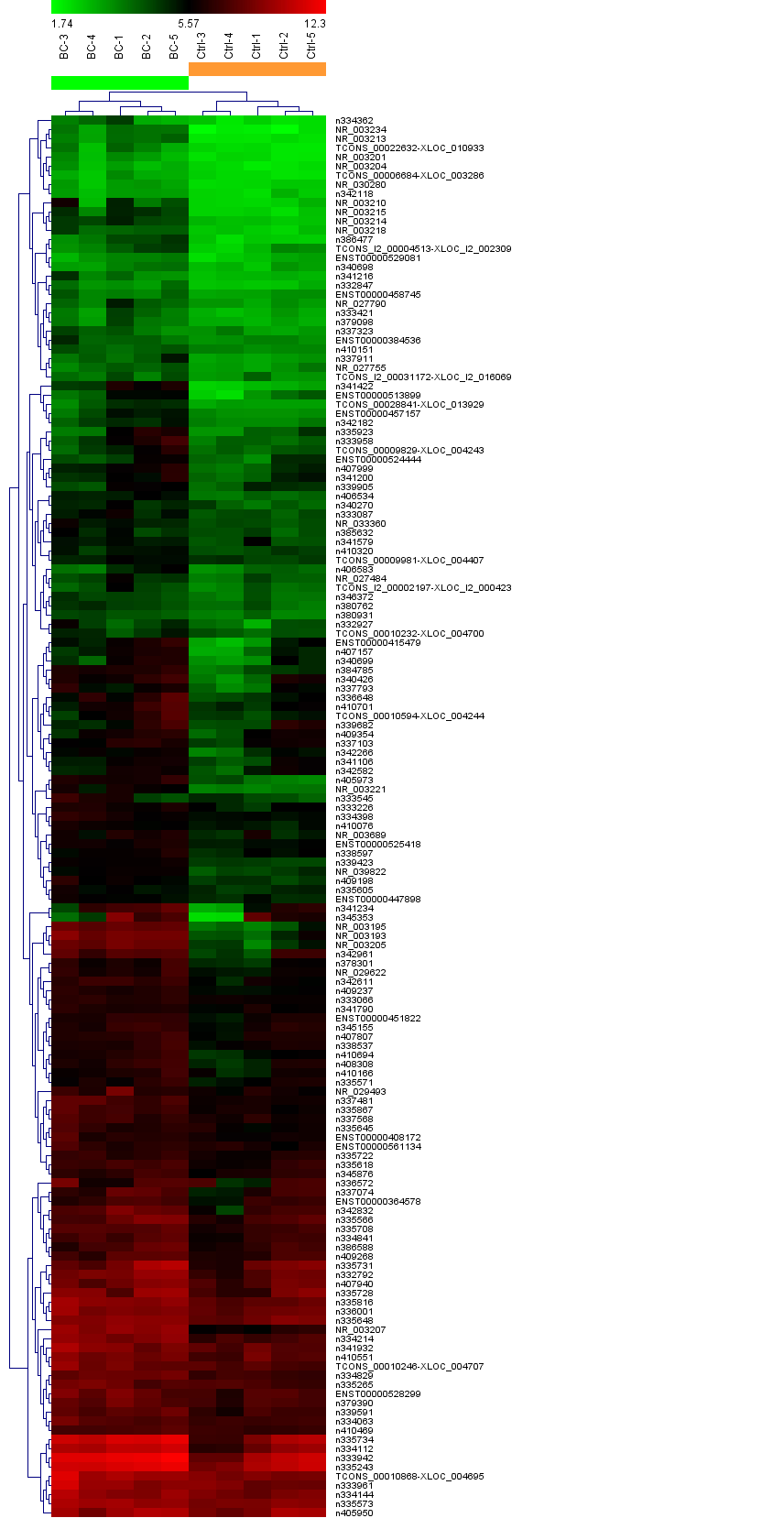


**B**

Figure.S2: Kaplan–Meier analysis of disease-free survival of breast cancer patients according to HER2 and HR status. Kaplan-Meier survival analysis of DFS rate in patients with (A) HER2− BC, (B) HER2+ BC, (C) HER2−HR+ BC, (D) HER2−HR− BC.


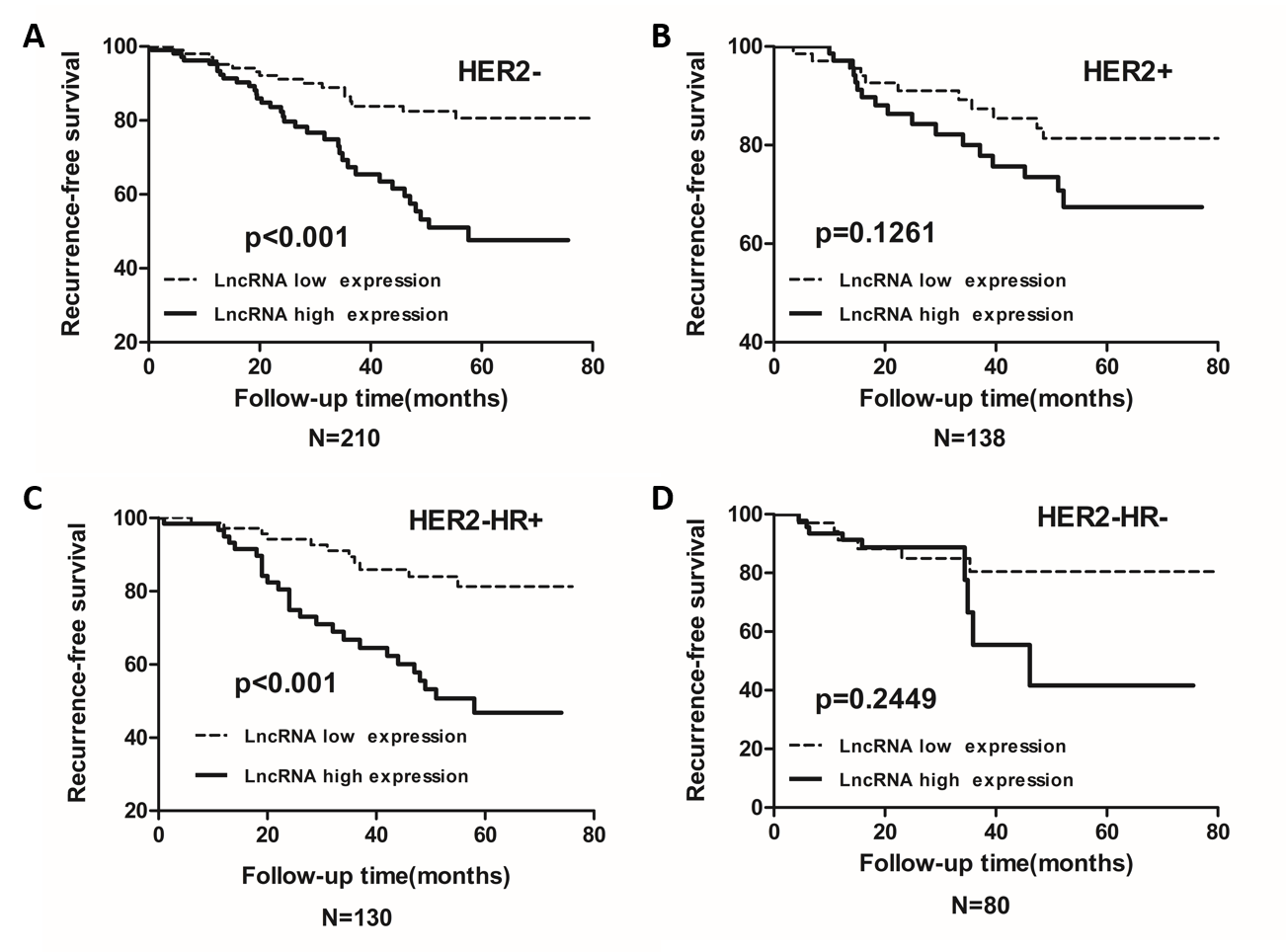


Table.S1: Significantly up-regulated lncRNAs determined by microarray (fold change ≥2.0 and P<0.05).

| Up-regulated lncRNAs | | |
| --- | --- | --- |
| **lncRNA** | **Log_2_ Fold Change (S/P)*** | **p-value** |
| n380673 | 2.84 | 0.020834 |
| NR_002712 | 2.53 | 0.020107 |
| n341420 | 2.44 | 0.031434 |
| n332338 | 2.42 | 0.032676 |
| n334545 | 2.38 | 0.04435 |
| ENST00000544591 | 2.26 | 0.042835 |
| n333883 | 2.22 | 0.04904 |
| n382161 | 2.2 | 0.029198 |
| n334594 | 2.17 | 0.031191 |
| n379706 | 2.15 | 0.02419 |
| n335494 | 2.03 | 0.014567 |

*S/P means metastatic axillary nodes (S) relative to corresponding tumor samples (P)
